# MEWS++: Enhancing the Prediction of Clinical Deterioration in Admitted Patients through a Machine Learning Model

**DOI:** 10.3390/jcm9020343

**Published:** 2020-01-27

**Authors:** Arash Kia, Prem Timsina, Himanshu N. Joshi, Eyal Klang, Rohit R. Gupta, Robert M. Freeman, David L Reich, Max S Tomlinson, Joel T Dudley, Roopa Kohli-Seth, Madhu Mazumdar, Matthew A Levin

**Affiliations:** 1 Institute for Healthcare Delivery Science, Department of Population Health Science and Policy, Icahn School of Medicine at Mount Sinai, New York, NY 10029, USA; 2 Department of Diagnostic Imaging, The Chaim Sheba Medical Center at Tel HaShomer, Sackler Faculty of Medicine, Tel Aviv University, Ramat Gan 52662, Israel; 3 Institute for Critical Care Medicine, Icahn School of Medicine at Mount Sinai, New York, NY 10029, USA; 4 Department of Anesthesiology, Perioperative and Pain Medicine, Icahn School of Medicine at Mount Sinai, New York, NY 10029, USA; 5 Department of Genetics and Genomics Sciences, Icahn School of Medicine at Mount Sinai, New York, NY 10029, USA; 6 Icahn Institute for Data Science and Genomic Technology, Icahn School of Medicine at Mount Sinai, New York, NY 10029, USA

**Keywords:** Failure to Rescue, Modified Early Warning Score, clinical deterioration, Machine Learning Classifiers, Unexpected Escalation

## Abstract

Early detection of patients at risk for clinical deterioration is crucial for timely intervention. Traditional detection systems rely on a limited set of variables and are unable to predict the time of decline. We describe a machine learning model called MEWS++ that enables the identification of patients at risk of escalation of care or death six hours prior to the event. A retrospective single-center cohort study was conducted from July 2011 to July 2017 of adult (age > 18) inpatients excluding psychiatric, parturient, and hospice patients. Three machine learning models were trained and tested: random forest (RF), linear support vector machine, and logistic regression. We compared the models’ performance to the traditional Modified Early Warning Score (MEWS) using sensitivity, specificity, and Area Under the Curve for Receiver Operating Characteristic (AUC-ROC) and Precision-Recall curves (AUC-PR). The primary outcome was escalation of care from a floor bed to an intensive care or step-down unit, or death, within 6 h. A total of 96,645 patients with 157,984 hospital encounters and 244,343 bed movements were included. Overall rate of escalation or death was 3.4%. The RF model had the best performance with sensitivity 81.6%, specificity 75.5%, AUC-ROC of 0.85, and AUC-PR of 0.37. Compared to traditional MEWS, sensitivity increased 37%, specificity increased 11%, and AUC-ROC increased 14%. This study found that using machine learning and readily available clinical data, clinical deterioration or death can be predicted 6 h prior to the event. The model we developed can warn of patient deterioration hours before the event, thus helping make timely clinical decisions.

## 1. Introduction

Timely detection of patient deterioration and prompt clinical intervention is key to lowering the potentially preventable morbidity and mortality among hospital inpatients [1,2]. All too often however, the early abnormalities and clinical signs that precede serious clinical deterioration may remain unidentified [3,4]. Studies on the identification of hospital patients at risk for clinical deterioration over last two decades have resulted in the development of prediction tools that use rule-based scoring [5,6,7,8]. The Modified Early Warning Score (MEWS), for example, incorporates physiological parameters including systolic blood pressure, pulse rate, respiratory rate, temperature, and level of consciousness. There are some limitations to this approach: (1) the schemas of these scores are usually defined manually; (2) alarm triggers rely on empirically chosen values; (3) the thresholds are usually set to capture the greatest percentage of clinically significant events, resulting in non-specific alerts that include a large number of false alarms. This creates an excess of warning notifications that can generate alarm fatigue [9,10,11]. Indeed, it has been shown that as the number of non-actionable alarms increases, the response time of providers increases as well [12]. Additionally, the usefulness of these systems is limited by inability to quantify the risk for decompensation and the lack of a defined time window for intervention.

Recent work by Bedoya et al. has confirmed the poor performance and minimal impact of implementing a traditional Early Warning Score [13]. However, machine learning approaches that use large Electronic Health Record (EHR) data can be trained to have good performance in predicting deterioration, exceeding that of traditional models [14,15]. We hypothesized that a machine learning model trained on a large dataset could have better performance than MEWS. We aimed for our model to predict escalation of care or death within the next 6 h. A six-hour prediction window was chosen based on clinical considerations such as the typical duration of nursing and physician shift length (8–12 h) and the desire to alert within a time frame that was both believable and actionable by the same care team that received the alert.

## 2. Patients and Methods

Institutional Research Board approval was obtained for this retrospective cohort study. Inclusion criteria were all adult inpatient admissions (age > 18) between July 2011 and July 2017. We excluded patients admitted to psychiatry, labor and delivery, and hospice units. This is due to low frequency of escalation or lack of adequate monitoring in these units. Patients were categorized into Major Diagnostic Categories (MDCs) derived from ICD-9 diagnostic codes [16].

We retrieved data via our institutional data warehouse from the following sources: admission-discharge-transfer (ADT) events; structured clinical assessments (e.g., nursing notes); physiologic data (e.g., vital signs); laboratory results; and automated electrocardiogram (ECG) results.

### 2.1. Phenotyping of Patient Deterioration

Most existing MEWS algorithms have been developed on cohorts of modest size (i.e., dozens to hundreds of patients). Adverse events were identified via either retrospective manual chart review or prospective data collection [6,17,18]. Given our intention to use a much larger cohort, we developed an automated phenotyping algorithm to identify escalation of care [19]. We decided a priori to base our algorithm on bed movement (ADT) data. Our base assumption was that bed movements were independent events. Using administrative data, each bed in the hospital was assigned to a generalized level of care such as floor, ICU, step-down, and so-on. We then applied a set of rules developed by authors MAL and RF to classify each bed transition as *expected* or *unexpected* (see Table S1 in the Supplement for the full list of phenotyping rules). *Expected* transitions were those between beds at the same level of care. *Unexpected* transitions were those to a higher level of care such as the ICU or step-down unit, or death.

A retrospective chart review was performed to validate the performance of the phenotyping algorithm. A random sample of 286 hospital encounters (1193 bed movements) was drawn from 157,984 hospital encounters. Authors MAL and RF reviewed the charts in consensus and classified each as a true positive, false positive, true negative or false negative. The review was done in rounds of 20–40 charts to tune the phenotyping algorithm. The final result showed sensitivity of 90.5%, specificity of 81.9%, and positive predictive value (PPV) of 82.1%.

### 2.2. Algorithms Evaluated

We compared a Random Forest (RF) algorithm to two additional machine learning algorithms: a linear Support Vector Machine (SVM) and Logistic Regression (LR). RF is a classifier that fits a number of decision trees on sub-samples of the dataset and uses averaging to improve the predictive accuracy and control over-fitting [20]. SVM is a classifier that attempts to maximize the linear distance between *p*-dimensional vectors representing instances of each class, where *p* is the number of features [21,22].

### 2.3. Defining Optimal Prediction Time

**Figure 1:**
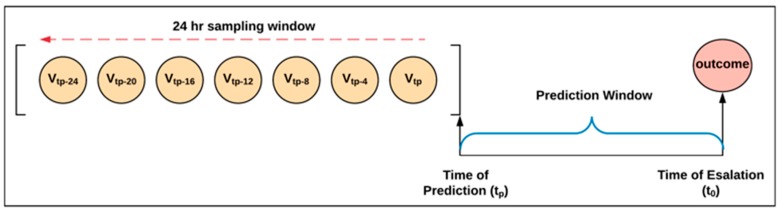
Prediction time and sampling window. t_0_ is the time of escalation, death, or discharge for patients with no event. Prediction time t_p_ is the time prior to t_0_ at which a prediction was generated. The sampling window is the 24-h period preceding the prediction time t_p_.

The time of escalation (*unexpected* transition) or death was defined as t_0_. For patients who never had an escalation of care (negative cases), the time of discharge was used as t_0._ Times of predictions, t_p,_ were defined as the time prior to t_0_ at which predictions were generated (Figure 1). Predictions were generated every two hours prior to the escalation event, with data sampled from the 24-h period prior to prediction (Figure 1). A time series was created by defining a sampling window as the 24-h period before the prediction time t_p_. Then, data were sampled every 4 h within the window, i.e., six times in 24 h (Figure 1). This frequency reflects the typical interval between vital sign measurements on a medical-surgical hospital unit. The result was a time series V = {V_1_, V_2_, … V_6_} with 6 measurements per 24-h sampling window for each feature. Missing values were imputed by using the median value of the variable over the entire cohort at the sampling time point [23].

Performance of the models were compared, and an optimal prediction time of 6 h was chosen based on clinical and operational considerations such as anticipated impact on nursing and clinician workload.

### 2.4. Data Encoding and Scaling

Categorical variables were one-hot-encoded. Continuous variables were scaled using the MLLib min-max scaler to be within the range (0,1) [24].

### 2.5. Resampling

The overall rate of *unexpected* events (escalation of care) was under 5%, versus 95% *expected* events. This large variance between the prevalence of the two classes can lead to over-fitting and under-performance [25]. We applied random under-sampling to remove instances of the majority class (*expected* events) until both classes were equally balanced.

### 2.6. Calculation of MEWS Score

MEWS score was calculated as described by Subbe et al. [6]. Briefly, four physiologic variables (systolic blood pressure, pulse rate, temperature, and respiratory rate) and one level of consciousness assessment were given a numeric weighting of 0–3 with the MEWS score calculated as the additive sum of all 5 sub-scores. A MEWS score of 2 was used as the cut-off based on the literature and by visual inspection of the sensitivity and specificity curves [6] (Supplementary Figure S1).

## 3. Model Development

All models were built using Python v.2.7.13, MLlib, and Spark 2.3 [26]. Plotly 2.0.6 was used for visualizations. The primary model developed was the RF model. We called this model MEWS++. We also evaluated the linear SVM and LR models. The default probability threshold of 0.5 was used for all models. Parameters for the models are listed in Supplementary Table S2. Briefly, the RF model used 500 trees, max depth of 10 and max bin of 32. Both SVM and LR models used 10 folds and 500 iterations. The regularization parameter for the SVM model was 0.1 and for the LR model it was 0.3. The Spark implementation of linear SVM has no slack parameter.

Feature selection and model tuning were performed on a training set consisting of 70% of the bed movement data. Ten-fold cross-validation was used for both feature selection and model development. The F_1_ score was used for best model selection. F_1_ is the harmonic average of the precision (PPV) and recall (sensitivity), calculated as:

(1) $$F_{1}=2*\frac{precision*recall}{precision+recall}$$

### 3.1. Feature Selection

Recursive feature elimination (RFE) was used as the feature selection approach. Feature selection was only performed only on the training set. A list of 89 variables was created based on review of relevant literature and clinician feedback, and were used to build an initial RF model (Supplementary Table S3). Then, using the Gini coefficient, the least important features were pruned [27]. All features were recursively tested in this fashion. The final list of variables chosen by RFE is listed in Supplementary Table S4.

### 3.2. Model Training

The final reduced feature set selected by RFE was then used to build a model, and this model was trained and cross-validated only on the training set. An average F_1_ score was computed for each internal validation model. The final model chosen for testing was the one with the largest average F_1_ score.

Under-sampling may result in loss of potentially useful information for defining the majority class, and thereby can compromise the model accuracy. In order to rule out such influence on RF/MEWS++ model performance, we performed 10 iterations of under-sampling on the majority class in the training data and compared the performance of resultant models with the main RF-based model. The results are shown in Supplementary Table S5. The standard deviations of all performance metrics ranged from 0.003 to 0.67, indicating that the final chosen RF model was not affected by under-sampling bias.

### 3.3. External Model Testing

The remaining thirty percent (30%) of the bed movement data were used exclusively as an independent test set for the final chosen RF model. PPV (precision), sensitivity (recall), specificity, F_1_ score and the Area Under the Receiver-Operator Curve (AUC) and Area Under the Precision-Recall Curve (AUPRC) were used as metrics.

## 4. Results

The raw cohort consisted of 96,645 patients with 157,984 hospital encounters and 244,343 bed movements. Under-sampling resulted in a training cohort of 15,818 bed movements. The test cohort consisted of 102,066 bed movements. Basic demographics of the training and test cohorts are shown in Table 1. The mean age was 63.4 years. Approximately half of the population was female (50.1%). The rate of unexpected escalation of care or death in the validation set was 3.4%. 56.8% escalated to an ICU, 41.9% to telemetry, 1.3% escalated to an intermediate care unit, and 0.7% died. There was a significant variation of unexpected escalation rate seen between MDC categories (Supplementary Figure S2). The respiratory, infectious, and circulatory groups showed the highest rates of escalation (9.2%, 5.9%, and 4.5%, respectively).

### Performance of Machine Learning Models at 6 h Prior to Escalation

**Figure 2:**
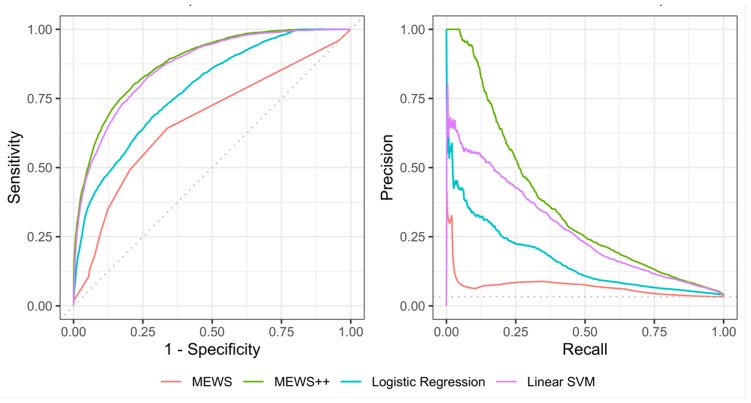
ROC and AUC PR Curves. Receiver Operating Characteristic (ROC) curves (left panel) and Precision-Recall curves (right panel) for the four models evaluated. MEWS++ (RF) performs better than other algorithms. LR—Logistic Regression, SVM = Support Vector Machine.

The model results are shown in Table 2. At 6 h prior to escalation, classical MEWS using a cutoff score of 2 had sensitivity of 64.5%, specificity of 66.6% and AUC of 0.67 (Table 2). MEWS++ (the RF model) had the best performance of all ML models and performed significantly better than classical MEWS, with sensitivity of 81.6%, specificity of 75.5%, and AUC of 0.85 (Table 2 and Figure 2). PPV also improved, with an AUPRC of 0.39 (Table 2 and Figure 2). Interestingly, while the difference between the ROC curves of linear SVM and the RF model was not significant (*p* = 0.16), superior performance of RF was seen by comparing the computed AUCPR and visual inspection of the precision recall (PR) curves (Figure 2). The AUCPR for the RF model was 36.2% vs. 28.7% for linear SVM, with no overlap of the 95% confidence intervals (Table 2).

**Figure 3:**
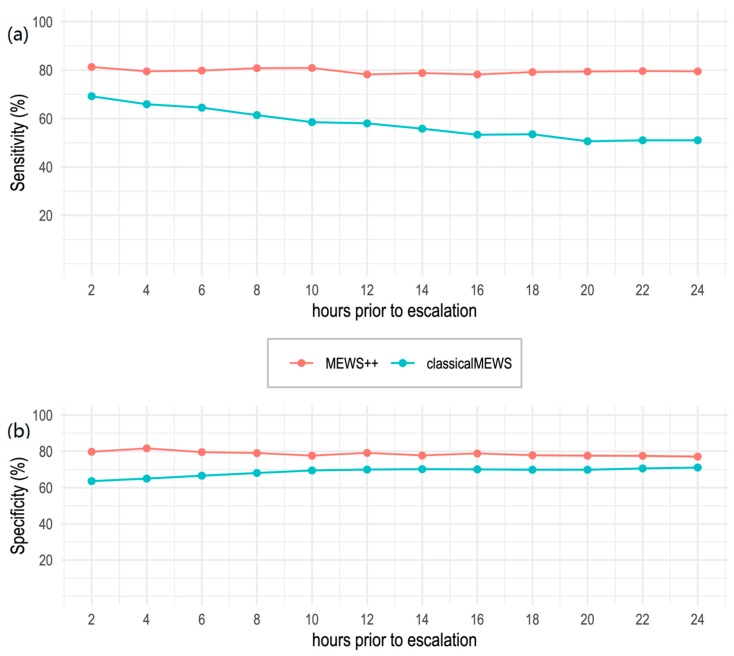
Comparison of 24-h performance of RF Model (MEWS++) vs. classical MEWS. Predictions were generated every 2 h for 24 h prior to escalation. A threshold of 2 was used for MEWS, and 0.5 (the default) for the RF model. (**a**) Sensitivity of MEWS begins to degrade after 4 h whereas sensitivity of MEWS++ remains stable. (**b**) Specificity of MEWS++ is consistently higher than MEWS.

The 24-h performance of MEWS++ vs. classical MEWS is shown in Figure 3. As can been seen, the RF model is stable over time, whereas the sensitivity of classical MEWS declined as the prediction time prior to escalation increased.

Since different MDCs showed different rates of unexpected escalation, a subgroup analysis was performed for the RF model for different MDCs. The five MDCs in which MEWS++ showed the best performance were Respiratory, Infectious, Digestive, Hepatobiliary/Pancreas, and Cardiovascular Diseases (Supplementary Figure S3).

## 5. Discussion

In this retrospective, single center study, we developed a ML model of patient deterioration (MEWS++) that significantly outperformed classical MEWS in predicting, 6 h in advance, clinical deterioration requiring transfer to a higher acuity unit, or death. The features utilized in the final model were variables that are readily available in all patient care areas, consisting mostly of demographics, vitals, lab results and physical exam findings.

MEWS++ improves on classical MEWS in several ways. The original MEWS algorithm was developed to identify patients at imminent risk of clinical deterioration, using a limited set of features [6]. We used a greatly expanded feature set, continuous value assessment that makes no a-priori assumptions about what constitutes a “normal” range, and time series forecasting where not only “point in time” measurements, but also prior data and trends, are incorporated into every prediction. The RF model demonstrated stable performance across a wide prediction window from 2 h to up to 24 h prior to the escalation of care. The chosen 6-h window gives enough time for intervention without straining clinical credibility. Similar to the “golden hour principle” that has been applied to a number of clinical conditions, including acute coronary syndrome, stroke and severe sepsis, a 6-h window could enable timely evidence-based interventions that improve outcomes for these patients [28,29,30].

One challenge with using ML models for clinical prediction is model interpretability [31,32]. It is desirable to present clinicians with a list of most important features. Such a list may build trust in the algorithm as well as provide clinical context for potential action. Ranking features by their Gini coefficient is one approach that can be applied to RF models [27,33]. The top features for MEWS++ are shown in Supplementary Figure S4. When applied to an individual patient, the list of features will vary. For example, the top 10 most important features for patient A may be pulse, BUN, age, systolic BP, bilirubin, diastolic BP, respiration rate, sodium, and lactate. The prediction for Patient B might have a very similar set of most important features, but instead of lactate have the INR. Thus, the features give individualized insight into why a prediction score might be high for a given patient.

Our approach shares similarity with the index developed by Rothman et al [8]. We used similar input data—nursing flowsheets, labs, vitals, and so forth—and similar sampling methodology. Where our approach differs was in the clinical endpoint used for model development and validation (escalation of care/death on the floor vs. 1-year mortality), and the algorithm employed (RF vs. LR). Although the Rothman index showed good performance for an LR model, we found that in our case the RF model performed better than LR. This may have been because of the large numbers of features [34].

While ML approaches based on regression and SVM have been evaluated to detect patient deterioration using only vital signs or in combination with laboratory test results, we found that an RF model performed better than SVM for our use case, with a much higher AUCPR (36.2% vs. 28.7%) [35,36,37,38]. This finding could be explained by the ability of RF to provide a non-parametric, hard to over-train model which is relatively robust to outliers and noise [39]. Therefore, we felt that the RF-based model was most suitable for this study.

### Limitations

There are several limitations to our approach. This was a single center, retrospective study. Our results may not be replicable at other institutions with different patient populations. The phenotyping algorithm used to identify unexpected escalations is based on purely administrative data with no clinical context. The algorithm cannot distinguish between an appropriate escalation to an ICU versus an unplanned emergent transfer—an example of the Frame Problem [40]. The assumption that bed movements are independent events and that prior bed movements do not influence future bed movements may be incorrect. Thus, our phenotype is not a perfect gold standard. This bias in the phenotyping is carried forward into the ML classifier, although the model does attenuate the bias. Also, we did not calibrate the prediction threshold [41,42]. Finally, another limitation of using ML methods is that scores become hard to interpret, as opposed to linear models where determining the contributors to a positive result are easily conceptualized [31]. Nonetheless, ML models may augment human intuition by finding hidden patterns in large datasets.

## 6. Conclusions

Using ML and a large database we have developed a predictive model called MEWS++ that has significantly better performance than the classic MEWS. MEWS++ can warn of patient deterioration 6 h prior to the event and thus help clinicians make timely interventions. Future models could be improved by incorporating additional data, such as more laboratory results, fluid intake and output, medication data, and free text from provider notes. Looking ahead, the success of using an ML model such as the one developed here as a clinical tool is contingent on its proper integration into healthcare system workflows. This work will require multidisciplinary collaboration between data scientists, clinicians, and hospital administrators, in order to fully realize the goal of improved clinical care.

## Figures and Tables

**Table 1:** Cohort demographics.

		Total *N* (%)	Training (%)	Test (%)	*p*-Value
Bed movements		117,884	15,818	102,066	
Bed movements per encounter		1.67 ± 1.15	1.33 ± 0.76	1.59 ± 0.99	
Unique Patients *		63,100	13,168	58,742	
Age	18–45	19,422 (16.5)	2107 (13.3)	17,315 (17.0)	<0.001
	45–65	40,942 (34.7)	5060 (32.0)	35,882 (35.2)	
	65–80	37,596 (31.9)	5266 (33.3)	32,330 (31.7)	
	>80	19,924 (16.9)	3385 (21.4)	16,539 (16.2)	
Gender	Female	58,345 (49.5)	7760 (49.1)	50,585 (49.6)	0.5
	Male	59,532 (50.5)	8057 (50.9)	51,475 (50.4)	
	Other	7 (0.0)	1 (0.0)	6 (0.0)	
Major Diagnostic Category (MDC)	Circulatory system	29,904 (25.4)	3930 (24.8)	25,974 (25.4)	<0.001
	Musculoskeletal system & connective tissue	12,521 (10.6)	1291 (8.2)	11,230 (11.0)	
	Nervous system	8767 (7.4)	1329 (8.4)	7438 (7.3)	
	Hepatobiliary/pancreas	7368 (6.3)	1223 (7.7)	6145 (6.0)	
	Respiratory system	7094 (6.0)	1190 (7.5)	5904 (5.8)	
	Infectious & parasitic	5762 (4.9)	1327 (8.4)	4435 (4.3)	
	Kidney & urinary tract	5474 (4.6)	723 (4.6)	4751 (4.7)	
	Endocrine/nutrition/metabolic	4207 (3.6)	513 (3.2)	3694 (3.6)	
	Ear, nose, mouth, and throat	2859 (2.4)	319 (2.0)	2540 (2.5)	
	Female reproductive system	2809 (2.4)	259 (1.6)	2550 (2.5)	
	Skin, subcutaneous tissue, breast	2459 (2.1)	236 (1.5)	2223 (2.2)	
	Other (MDCs with ≤ 2% occurrence)	28,660 (24.3)	3478 (22)	25,182 (24.7)	
Overall length of stay at hospital	≤5 days	52,087 (44.2)	5410 (34.2)	46,677 (45.7)	<0.001
	5–12 days	35,210 (29.9)	4876 (30.8)	30,334 (29.7)	
	12–42 days	26,753 (22.7)	4482 (28.3)	22,271 (21.8)	
	>42 days	3834 (3.3)	1050 (6.6)	2784 (2.7)	
Length of stay by hospital unit	≤24 h	52,932 (44.9)	6699 (42.4)	46,233 (45.3)	<0.001
	1–3 days	35,748 (30.3)	4865 (30.8)	30,883 (30.3)	
	3–7 days	20,916 (17.7)	2833 (17.9)	18,083 (17.7)	
	>7 days	8288 (7.0)	1421 (9.0)	6867 (6.7)	
Length of stay in the ICU	≤24 h	2805(28.8)	198 (27.1)	2607 (29.0)	0.36
	1–3 days	4048 (41.6)	322 (44.1)	3726 (41.4)	
	3–7 days	1928 (19.8)	134 (18.4)	1794 (19.9)	
	>7 days	947 (9.7)	76 (10.4)	871 (9.7)	

* Some patients appeared in both training and test sets because the data were split on bed movements, not patients.

**Table 2:** Model performance metrics.

Model	Sensitivity, % (95% CI)	Specificity, % (95% CI)	Accuracy, % (95% CI)	PPV, % (95% CI)	F1 Score	ROC (95% CI)	AUC PR (95% CI)	*p*-Value *
Random Forest (MEWS++)	78.9 (77.6–80.1)	79.1 (78.9–79.3)	79.1 (78.9–79.3)	11.5 (11.1–11.9)	0.2	87.9 (87.4–88.4)	36.2 (34.7–37.7)	<0.0001
Linear SVM	79.0 (77.6–80.3)	77.9 (77.6–78.1)	77.9 (77.7–78.2)	11.0 (10.6–11.4)	0.19	87.3 (86.8–87.9)	28.7 (27.2–30.2)	<0.00010.16 **
LR	61.4 (59.8–63.0)	78.5 (78.3–78.8)	77.9 (77.7–78.2)	9.0 (8.6–9.4)	0.16	79.1 (78.4–79.8)	17.2 (16.0–18.5)	<0.0001
MEWS Score	64.2 (62.7–65.7)	66.2 (66.0–66.5)	66.2 (65.9–66.4)	6.1 (5.9–6.4)	0.11	66.7 (65.9–67.6)	7.0 (6.2–7.8)	

* *p*-value for difference between AUC ROC for respective ML model and MEWS Score. ** *p*-value = 0.16 for Random Forest vs. Linear SVM. AUCPR—Area Under Precision Recall Curve, LR—Linear Regression, SVM—Support Vector Machine, ROC—Receiver Operating Characteristic.

## Supplementary Materials

The following are available online at https://www.mdpi.com/2077-0383/9/2/343/s1, Figure S1: Sensitivity and Specificity of Classical MEWS at Different Thresholds; Figure S2: Rate of Escalation by MDC; Figure S3: Sub-group analysis of MEWS++ Performance by Major Diagnostic Category (MDC); Figure S4: Top Features contributing to model, ranked by Gini coefficient; Table S1: Phenotyping Rules; Table S2: Model Parameters; Table S3: Initial List of Features (Variables); Table S4: Final List of 36 Features (Variables) Chosen by Recursive Feature Elimination; Table S5: Results of 10-fold under-sampling of training data on final RF model performance.
