# Supplementary material for: MEWS++: Enhancing the Prediction of Clinical Deterioration in Admitted Patients through a Machine Learning Model
Source: J Clin Med. 2020 Jan 27;9(2):343. doi: 10.3390/jcm9020343 (PMC7073544; doi:10.3390/jcm9020343)
Supplement: Supplementary file 1 [file jcm-09-00343-s001.pdf]

Supplementary Material for MEWS++: Automated Phenotyping and Machine Learning to Predict Clinical Deterioration during Hospital Admissions

Phenotyping rules used to automatically identify escalation of care are shown in Table 1. Each bed location in the hospital was assigned a level of care. Beds/locations excluded from the phenotyping (e.g. hospice, psychiatric unit) were labeled **Exclude**. Placeholder beds used by the bed management system (e.g. pre-admissions, bed reservations) were labeled **Skip**. Every combination of bed movement/transfer during a given patient's admission was then labeled as *Expected, Unexpected,* or *Exclude*. *Unexpected* transfers were considered escalation of care.

Table S1. Phenotyping Rules.

**Current bed level of care**	**Next bed level of care**	**Label**
Exclude	Routine Discharge	*Exclude*
Exclude	Exclude	*Exclude*
Exclude	Floor	*Exclude*
Exclude	ICU	*Exclude*
Exclude	OR	*Exclude*
Exclude	Step down	*Exclude*
Exclude	Telemetry	*Exclude*
Floor	Routine Discharge	*Expected*
Floor	Exclude	*Exclude*
Floor	Floor	*Expected*
Floor	ICU	*Unexpected*
Floor	OR	*Expected*
Floor	PACU	*Exclude*
Floor	Step down	*Unexpected*
Floor	Telemetry	*Unexpected*
Floor	Death	*Unexpected*
ICU	Routine Discharge	*Expected*
ICU	Exclude	*Exclude*
ICU	Floor	*Expected*
ICU	ICU	*Expected*
ICU	OR	*Expected*
ICU	PACU	*Exclude*
ICU	Step down	*Expected*
ICU	Telemetry	*Expected*
ICU	Death	*Expected*
Skip	Routine Discharge	*Exclude*
Skip	Exclude	*Exclude*
Skip	Floor	*Exclude*
Skip	ICU	*Exclude*
Skip	OR	*Exclude*
Skip	PACU	*Exclude*
Skip	Step down	*Exclude*
Skip	Telemetry	*Exclude*

OR	Routine Discharge	*Exclude*
OR	ED	*Exclude*
OR	Exclude	*Exclude*
OR	Floor	*Expected*
OR	ICU	*Expected*
OR	OR	*Exclude*
OR	PACU	*Expected*
OR	Step down	*Expected*
OR	Telemetry	*Expected*
OR	Death	*Unexpected*
PACU	Routine Discharge	*Expected*
PACU	Floor	*Expected*
PACU	ICU	*Unexpected*
PACU	OR	*Unexpected*
PACU	PACU	*Expected*
PACU	Step down	*Expected*
PACU	Telemetry	*Expected*
PACU	Death	*Expected*
Step down	Routine Discharge	*Expected*
Step down	Exclude	*Exclude*
Step down	Floor	*Expected*
Step down	ICU	*Unexpected*
Step down	OR	*Expected*
Step down	Step down	*Expected*
Step down	Telemetry	*Expected*
Step down	Death	*Unexpected*
Telemetry	Routine Discharge	*Expected*
Telemetry	Exclude	*Exclude*

OR = Operating Room

Table S1 Model Parameters.

**Model**	**Parameters**
Random Forest	*Number of trees=500*<br>*Max Depth = 10*<br>*Max Bin =32*<br>*Subset strategy = "one-third"*
Linear SVM	*Number of folds = 10*<br>*Maximum number of iterations = 500*<br>*Depth for treeAggregate = 10*<br>*Regularization parameter = 0.1*<br>*Convergence tolerance for iterative algorithms = 0.001*<br>*Standardization = True*
Logistic Regression	*Number of folds = 10*<br>*Maximum number of iterations = 500*<br>*ElasticNet mixing parameter = 0.2*<br>*Regularization parameter = 0.3*<br>*Fit an intercept = True*

Table S3 Initial List of Features (Variables)

**Feature/Variable**	**Data Type**	**Data Source**
Age	Float	ADT System
Gender	String	ADT System
Transition type	String	ADT System
Unit type	String	ADT System
Unit LOS	Float	*Derived from (Unit_Transition_time - Unit_Admit_time)*
Admission source	String	ADT System
Admission type	String	ADT System
Specialty unit	String	ADT System
Living will indicator	String	ADT System
Oral temperature	Vector (Float)	Epic
Pulse	Vector (Float)	Epic
Respirations	Vector (Float)	Epic
Systolic blood pressure	Vector (Float)	Epic
Diastolic blood pressure	Vector (Float)	Epic
Weight	Vector (Float)	Epic
Height	Float	Epic
O2 saturation	Vector (Float)	Epic
Fio2	Vector (Float)	Epic
High tidal volume	Vector (Float)	Epic
Inspiratory tidal volume	Vector (Float)	Epic
Low tidal volume	Vector (Float)	Epic
CVP	Vector (Float)	Epic
CPAP	Vector (Float)	Epic
MAP mmHg	Vector (Float)	Epic
Expiratory tidal	Vector (Float)	Epic
I e ratio	Vector (Float)	Epic
PIP cm H_2O	Vector (Float)	Epic
PVRI	Vector (Float)	Epic
SvO2	Vector (Float)	Epic
Activity	Vector (String)	Epic
Bilateral breath sounds	Vector (String)	Epic
Bowel sounds all quadrants	Vector (String)	Epic
Cardiac regularity	Vector (String)	Epic
Generalized edema	Vector (String)	Epic
Level of consciousness	Vector (String)	Epic
Non surgical wound present	Vector (String)	Epic
Pressure ulcer poa	Vector (String)	Epic
Pressure ulcer pout	Vector (String)	Epic
Respiratory pattern	Vector (String)	Epic
Telemetry cardiac monitor	Vector (String)	Epic
Tenderness	Vector (String)	Epic
Aprdrg soi	String	Epic
Premature ventricular count	Vector (Float)	MUSE
Qt	Vector (Float)	MUSE
Num of vent ect	Vector (Float)	MUSE

Min heart rate	Vector (Float)	MUSE
Avg heart rate	Vector (Float)	MUSE
Num of supravent runs	Vector (Float)	MUSE
Num of qrs compl	Vector (Float)	MUSE
Num of vent bigeminal cycles	Vector (Float)	MUSE
Num of supravent isolated beats	Vector (Float)	MUSE
Vent rate	Vector (Float)	MUSE
Num of vent isolated beats	Vector (Float)	MUSE
Num of supravent couplets	Vector (Float)	MUSE
Num of vent beats in runs	Vector (Float)	MUSE
Atrial rate	Vector (Float)	MUSE
Max heart rate	Vector (Float)	MUSE
Num of vent couplets	Vector (Float)	MUSE
Qtc	Vector (Float)	MUSE
Num of vent run	Vector (Float)	MUSE
QRS duration	Vector (Float)	MUSE
P-R interval	Vector (Float)	MUSE
P axis	Vector (Float)	MUSE
Num of supravent ectopics	Vector (Float)	MUSE
Base-excess abg	Vector (Float)	Lab Information System
Base-excess vbg	Vector (Float)	Lab Information System
Bilirubin direct	Vector (Float)	Lab Information System
Bilirubin total	Vector (Float)	Lab Information System
Bnp	Vector (Float)	Lab Information System
Bun	Vector (Float)	Lab Information System
Ckmb	Vector (Float)	Lab Information System
Cl	Vector (Float)	Lab Information System
Cpk	Vector (Float)	Lab Information System
Creatinine	Vector (Float)	Lab Information System
Gfr estimate	Vector (Float)	Lab Information System
Hco3abg	Vector (Float)	Lab Information System
Hco3vbg	Vector (Float)	Lab Information System
Hematocrit	Vector (Float)	Lab Information System
Hemoglobin	Vector (Float)	Lab Information System
Hemoglobina1c	Vector (Float)	Lab Information System
INR	Vector (Float)	Lab Information System
K+	Vector (Float)	Lab Information System
Lactate	Vector (Float)	Lab Information System
Na	Vector (Float)	Lab Information System
Pct	Vector (Float)	Lab Information System
pH art	Vector (Float)	Lab Information System
Platelets	Vector (Float)	Lab Information System
Troponin-I	Vector (Float)	Lab Information System
Wbc	Vector (Float)	Lab Information System

Table S2 Final List of 36 Features (Variables) Chosen by Recursive Feature Elimination

**Feature/Variable**	**Data Type**	**Data Source**
Age	Float	ADT Data
Unit Type	String	ADT Data
Unit LOS	Float	Derived from (Unit_Transition_time - Unit_Admit_time)
Specialty Unit	String	ADT System
Admit SOURCE	String	ADT System
Admit TYPE	String	ADT System
WBC	Vector (Float)	Lab Information System
BUN	Vector (Float)	Lab Information System
Base Excess (ABG)	Vector (Float)	Lab Information System
HCO3(ABG)	Vector (Float)	Lab Information System
Creatinine	Vector (Float)	Lab Information System
Cl	Vector (Float)	Lab Information System
Lactate (ABG)	Vector (Float)	Lab Information System
Hematocrit	Vector (Float)	Lab Information System
Platelet Count	Vector (Float)	Lab Information System
INR	Vector (Float)	Lab Information System
Na	Vector (Float)	Lab Information System
Hemoglobin	Vector (Float)	Lab Information System
K+	Vector (Float)	Lab Information System
Bilirubin Total	Vector (Float)	Lab Information System
GFR Estimate	Vector (Float)	Lab Information System
Lactate (VBG)	Vector (Float)	Lab Information System
MAP MMHG	Vector (Float)	EHR (Epic)
Pulse Rate	Vector (Float)	EHR (Epic)
O2 Saturation	Vector (Float)	EHR (Epic)
Diastolic Blood Pressure	Vector (Float)	EHR (Epic)
Weight	Vector (Float)	EHR (Epic)
Systolic Blood Pressure	Vector (Float)	EHR (Epic)
Temperature (Oral)	Vector (Float)	EHR (Epic)
Height	Vector (Float)	EHR (Epic)
Respiratory Rate	Vector (Float)	EHR (Epic)
Level of Consciousness	Vector (String)	EHR (Epic)
Cardiac Regularity	Vector (String)	EHR (Epic)
Level of Activity	Vector (String)	EHR (Epic)
Respiratory Pattern	Vector (String)	EHR (Epic)
Tenderness	Vector (String)	EHR (Epic)

Table S3 Results of 10-fold under-sampling of training data on final RF model performance

	Sensitivity (%)	Specificity (%)	Accuracy (%)	F1 Score	ROC AUC (%)	AUC PR (%)
**iter1**	81.0 (79.7-82.3)	79.2 (78.9-79.4)	79.2 (79.0-79.5)	0.21	88.5 (88.0-89.1)	38.3 (36.7-39.9)
**iter2**	79.6 (78.2-81.0)	79.6 (79.4-79.9)	79.6 (79.4-79.9)	0.21	88.4 (87.8-88.9)	37.7 (36.0-39.3)
**iter3**	79.5 (78.2-80.9)	79.8 (79.5-80.0)	79.8 (79.5-80.0)	0.21	88.3 (87.8-88.9)	38.4 (36.7-40.0)
**iter4**	80.0 (78.6-81.3)	79.2 (79.0-79.5)	79.2 (79.0-79.5)	0.21	88.4 (87.8-88.9)	37.5 (35.8-39.1)
**iter5**	78.7 (77.3-80.0)	79.7 (79.5-80.0)	79.7 (79.5-80.0)	0.21	88.2 (87.7-88.7)	37.7 (36.0-39.3)
**iter6**	79.2 (77.8-80.5)	79.6 (79.3-79.8)	79.6 (79.3-79.8)	0.21	88.2 (87.7-88.8)	37.9 (36.3-39.5)
**iter7**	80.1 (78.8-81.5)	79.5 (79.2-79.7)	79.5 (79.2-79.7)	0.21	88.3 (87.8-88.9)	37.9 (36.2-39.5)
**iter8**	79.5 (78.1-80.9)	79.9 (79.7-80.2)	79.9 (79.7-80.2)	0.21	88.3 (87.7-88.8)	37.2 (35.6-38.8)
**iter9**	79.9 (78.5-81.3)	79.1 (78.9-79.4)	79.1 (78.9-79.4)	0.2	88.3 (87.7-88.8)	37.1 (35.5-38.8)
**iter10**	80.6 (79.3-81.9)	79.2 (78.9-79.4)	79.2 (79.0-79.5)	0.21	88.4 (87.9-88.9)	37.4 (35.7-39.0)
**Mean**	79.8	79.5	79.5	0.21	88.3	37.7
**StdDev**	0.67	0.29	0.29	0.003	0.095	0.43
**Final Model**	78.9	79.1	79.1	0.2	87.9	36.2

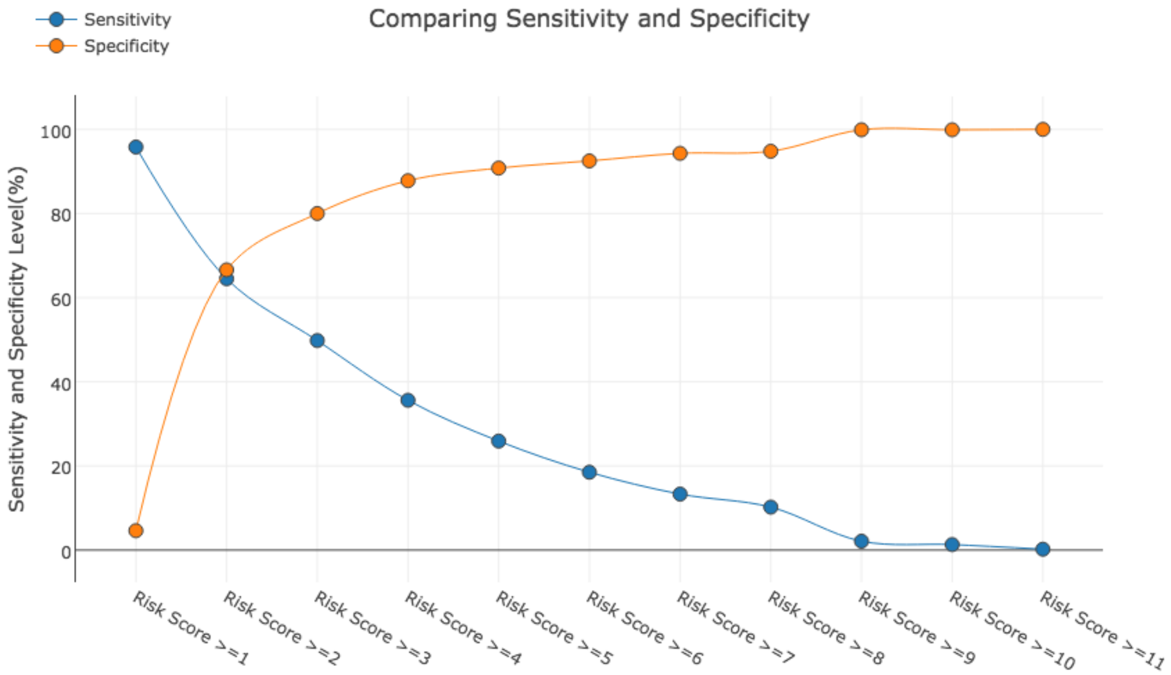

Figure S1 Sensitivity and Specificity of Classical MEWS at Different Thresholds

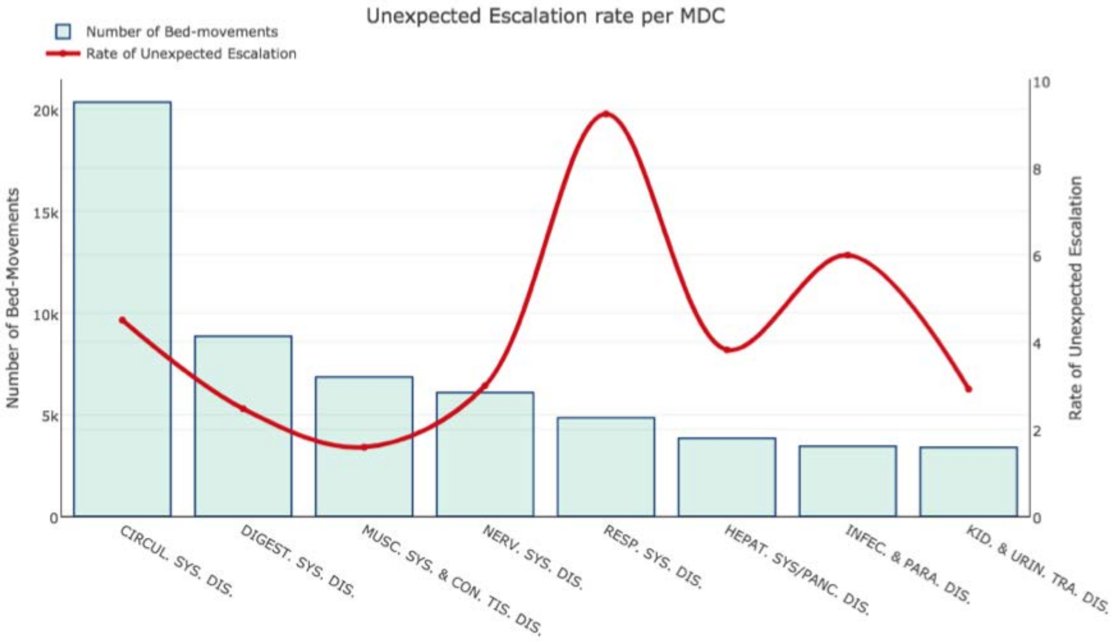

Figure S2 Rate of Escalation by MDC

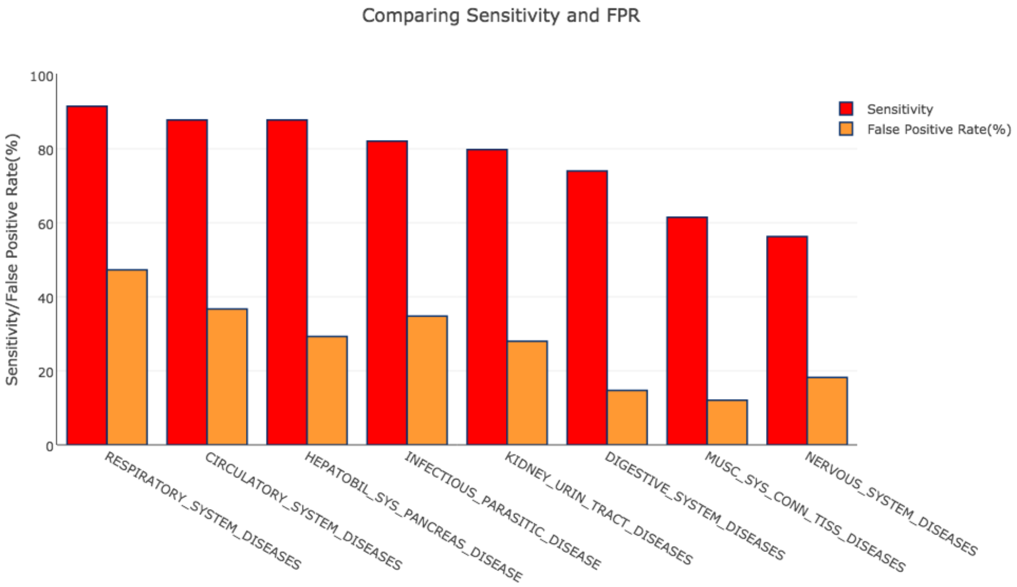

Figure S3 Sub-group analysis of MEWS++ Performance by Major Diagnostic Category (MDC)

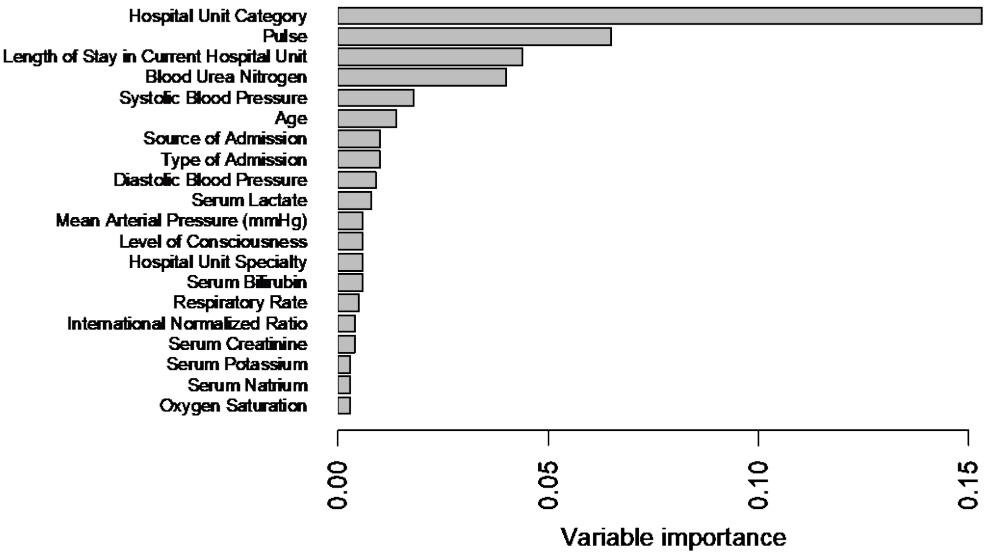

Figure S4 Top Features contributing to model, ranked by Gini coefficient
